# Identification and Localization of Track Circuit False Occupancy Failures Based on Frequency Domain Reflectometry

**DOI:** 10.3390/s20247259

**Published:** 2020-12-18

**Authors:** Tiago A. Alvarenga, Augusto S. Cerqueira, Luciano M. A. Filho, Rafael A. Nobrega, Leonardo M. Honorio, Henrique Veloso

**Affiliations:** 1 Electrical Engineering Department, Federal University of Juiz de Fora, Juiz de Fora 36036-900, MG, Brazil; tiago.araujo@engenharia.ufjf.br (T.A.A.); augusto.santiago@ufjf.edu.br (A.S.C.); luciano.ma.filho@gmail.com (L.M.A.F.); rafael.nobrega@ufjf.edu.br (R.A.N.); 2 MRS Logística, Juiz de Fora 36060-010, MG, Brazil; henrique.Veloso@mrs.com.br

**Keywords:** track circuit failures, frequency domain reflectometry, non-linear regression

## Abstract

Railway track circuit failures can cause significant train delays and economic losses. A crucial point of the railway operation system is the corrective maintenance process. During this operation, the railway lines have the circulation of trains interrupted in the respective sector, where traffic restoration occurs only after completing the maintenance process. Depending on the cause and length of the track circuit, identifying and solving the problem may take a long time. A tool that assists in track circuit fault detection during an inspection adds agility and efficiency in its restoration and cost reduction. This paper presents a new method, based on frequency domain reflectometry, to diagnose and locate false occupancy failures of track circuits. Initially, simulations are performed considering simplified track circuit approximations to demonstrate the operation of the proposed method, where the fault position is estimated by identifying the null points and through non-linear regression on signal amplitude response. A field test is then carried out in a track circuit approximately 1500 m long to validate the proposed method. The results show that the proposed method can identify and estimate the fault location due to a short circuit between rails with high accuracy.

## 1. Introduction

The economic demand and the growing necessity to transport goods and people have raised railways’ availability and safety demands. One way to optimize the track’s operationality is to provide fast and assertive inspections in its components. The most critical component is the signaling and control system, named Track Circuit (TC) [1]. The TC is a series of circuits installed over the railway that measures the shortages between tracks continuity between circuits. Therefore it is a device of vast importance, which can be employed to locate trains in a stretch and find track breaks.

Occurrences in the TC are related to the railway or to the circuit itself. The most common causes are false occupancy, poor track bed, shortage to the ground or between tracks and broken circuit or track, among others [2]. Therefore, any nonconformity in the TC is a critical operational issue and must be investigated and corrected before that specific railway section became functional again. Delays in finding and fixing the problem may disrupt the railway’s services and must be quickly corrected.

Two common and critical issues in almost every railway are broken and shorted track circuits. Such occurrences cause discontinuities on the TC that do not indicate or indicates false railway occupancy, respectively. Although simple circuit analyzers can quickly detect both scenarios [3], this equipment fails to identify false negative occurrences and does not provide the exact location of the failure. Considering that each TC section may have hundreds of meters, the search for the fault location consumes resources and time, generating economic losses.

Considering TC inspections and nonconformity detection, paper [4] compares the difference between traditional approaches and new methodologies such as ultrasonic inspections and risk management. It concludes that the main drawback of these new approaches is that they are not able to provide the location of a broken rail and their inspection time tends to be long since it depends on an inspection vehicle to run through the tracks [5], generating an extra railway occupancy for maintenance.

Paper [6] uses a neuro-fuzzy system for fault detection and diagnosis in TC. Although it has presented good simulated results, the approach does not indicate the fault location. Paper [7] uses a Dempster–Shafer classifier fusion and, although it provides adequate results, they were completely based on simulation, and the approach is specific for just one type of Alternating Current (AC) TC. Paper [8] has used the track current along with a neural network classifier to diagnose faults, but the approach does not indicate the fault location.

Methods regarding track circuits are based on collected measurements by trains [7,9] or by spreading sensors [10] through the railway. These two approaches face practical problems such as the TC block for train circulation in faults and the high cost related to the spread sensors throughout the railway line.

In [11], a study was presented analyzing the feasibility of applying the Time Domain Reflectometry (TDR) technique, to identify broken rail on railway lines. The proposed system would be embedded in the locomotive and from the TDR techniques, would act in the identification of the rail break as it went along the line. To be viable, the system would need to ensure a detection range greater than the distance a train in normal cruising conditions expended to brake the composition and thus avoid possible accidents. It would act in conjunction with Communications-Based Train Control (CBTC) based lines [12], which can perform rail traffic control without the existence of TC, but the CBTC system alone cannot perform the detection of broken rails. Through the simulations, the author points out to be viable the identification of broken rail at a distance of 1 to 2 miles, depending on the climatic conditions of the track.

The article sought to determine whether the demonstrated concept was technically feasible and whether further research might be necessary. In order to carry out field tests, it would be necessary to develop a complex system of coupling between the device and the locomotive. It would be necessary to evaluate the mechanical design and safety issues of the locomotive, which makes it very difficult to implement the proposal. Another attenuating factor is the limitation of the energy to be applied to the pulse in order to avoid structural damage due to interactions with the electrical components on the track, which could reduce the range of the system.

This paper, in a certain way, served as inspiration for the application of reflectometry to treat problems in the railway lines. However, it shows great distinction with the methods proposed in work.

There are applications, such as [13], which seeks to detect broken rail by monitoring TC voltage and transmitting information to the train through a radio frequency system. This proposed tool requires an amount of equipment to be installed by TC, as well as communication systems with trains. The main purpose is to avoid derailment due to the disruption of tracks. Besides needing a large number of equipment to be implemented, this proposal differs from the methods proposed in this work.

In [14] a method of detecting broken rails using acoustic transducers mounted beside the rail was proposed. Here also consists of a tool to prevent derailment due to the breaking of rails. This proposal demand for numerous equipment to be installed next to the railway increases the costs of installation and maintenance.

The most common approach in reflectometry is to inject an electromagnetic signal into the tracks and analyze its reflection. By processing information about the injected and reflected signals, it is possible to retrieve several valuable information regarding the problem, including its exact location. Reflectometry consists of a qualitative analysis of the reflected wave component of an electromagnetic signal propagating through the System Under Test (SUT) [15]. The reflected wave transcribes some information of the medium that one wants to characterize. The reflectometry is widespread in several sectors in a wide range of applications [16,17,18]. Nevertheless, the use of Frequency Domain Reflectometry (FDR) for TC fault location is not explored in the literature.

Therefore, this paper presents a new method based on FDR to diagnose and locate track circuits’ false occupancy failures caused by a short circuit between rails or a due to a discontinuity problem (e.g., broken rail or rail joint discontinuity problem). The proposed method uses the equipment connected to the faulty circuit and identifies both problem and location using FDR.

The rest of this paper is organized as follows. In Section 2, the track circuit basic concepts are presented. In Section 3, the wave propagation in the track circuit is discussed. Section 4 presents the proposed method for track circuit fault identification and location. The implemented system and the field test performed to validate the proposed method are presented in Section 5. In Section 6, a discussion about the field test result is presented. The conclusions of this paper are given in Section 7.

## 2. Railway Track Circuit

The railway signaling system was started based on controlling the track sections where a locomotive’s presence was detected. Thus the control of local traffic was carried out according to the flow of trains. The primary system used to identify the train for this task was the TC system [19,20,21]. TC is an electrical circuit created through the rails with the primary target of detecting trains’ presence in the corresponding stretch in which it is being applied [19,20]. It can also detect rails’ breaking due to its working principle, where it acts when circuit interruption occurs [22].

**Figure 1:**
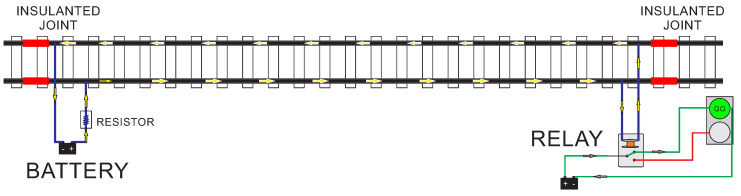
Example of a DC track circuit un-occupied.

In general, TC is based on the subdivision of a railway line into numerous circuits by installing insulating joints that electrically disconnect the rails building isolated blocks [19,20]. The operation principle is based on the transmission of an electrical signal from one edge to the other. Figure 1, presents a simplified representation of TC powered by Direct Current (DC). It indicates that the section is unoccupied because the current supplied by the battery and limited by a resistance runs to the other edge where it energizes a track relay.

**Figure 2:**
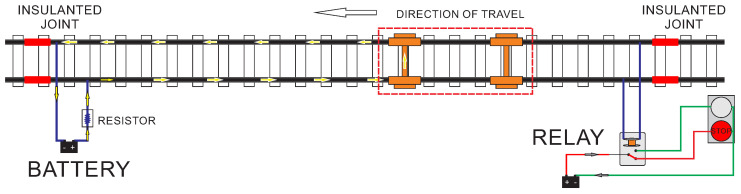
Example of a DC track circuit occupied by a train.

If for some reason, the relay is not energized, the section is now indicated as occupied. Figure 2 illustrates an example, where the TC is occupied by the presence of a train. The current supplied by the battery drive through the rail and returns through the contact established with the wheels of the train [19,20]. As the relay is no longer energized, the section is indicated as occupied. When a rail break occurs, the track’s electrical discontinuity is indicated as the stretch’s occupation.

The AC TC is energized by an AC voltage source in the range of audio frequencies (80 Hz up to 10 kHz), and the relay is arranged to detect the selected frequency and ignore the DC and AC traction frequency signals.

Some of the problems that can cause TC failures, resulting in occupied track sections, are listed below:

- a broken wire connecting the source or the relay on the running rails;
- an electrical discontinuity in a rail joint;
- a broken rail;
- a failure on the power supply;
- a short across the rails;
- a short between adjacent track circuit sections.

## 3. Wave Propagation Along Rail Track

**Figure 3:**
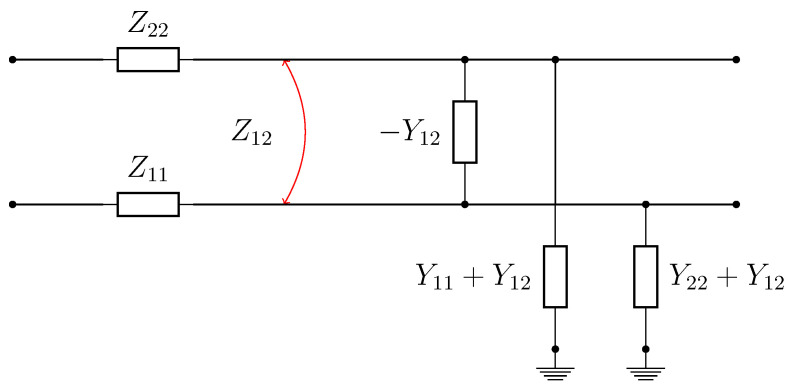
Two-wire Transmission Line (TL) model with Earth return.

In order to analyze the wave propagation in the TC, it can be modeled as a multi-conductor transmission line (MTL) [23,24]. Figure 3 shows a model for a two-wire line with earth return, which can be used as an equivalent representation of a section of a single rail track. In Figure 3, $z_{11}$ and $z_{22}$ are the self impedances per unit length, $y_{11}$ and $y_{22}$ are the self admittances per unit length, $z_{12}$ is the mutual impedance per unit length between the lines and $y_{12}$ is the mutual admittance per unit length between the lines.

The voltages and currents along a line are calculated by the transmission equations

(1) $$\frac{dV}{dx}=-\mathrm{ZI},$$

and

(2) $$\frac{dI}{dx}=-\mathrm{YV},$$

where V and I are column vectors of the track phase voltage and current. The Z and Y are the impedance and admittance matrices.

After deriving both sides and combining (1) and (2) the distance-dependent voltages and currents are related by the impedance and admittance matrices such that:

(3) $$\frac{d^{2}V}{dx^{2}}=ZYV=PV$$

and

(4) $$\frac{d^{2}I}{dx^{2}}=YZI=P^{t}I,$$

where

$$\begin{array}{c} V=\left[\begin{array}{c} V_{1}\\ V_{2} \end{array}\right],I=\left[\begin{array}{c} I_{1}\\ I_{2} \end{array}\right],Z=\left[\begin{array}{cc} z_{11} & z_{12}\\ z_{12} & z_{22} \end{array}\right],Y=\left[\begin{array}{cc} y_{11} & y_{12}\\ y_{12} & y_{22} \end{array}\right] \end{array}$$

and

$$\begin{array}{c} P=\left[\begin{array}{cc} z_{11}y_{11}+z_{12}y_{12} & z_{11}y_{12}+z_{12}y_{22}\\ z_{12}y_{11}+z_{22}y_{12} & z_{12}y_{12}+z_{22}y_{22} \end{array}\right]. \end{array}$$

The propagation constant $\gamma_{d}$ and characteristic impedance $Z_{0}$ are calculated by:

(5) $$\gamma_{d}=\sqrt{\mathrm{ZY}}$$

and

(6) $$Z_{0}=\sqrt{\frac{Z}{Y}},$$

where are variable in the frequency function. The problem can be simplified if spacial symmetry is assumed in the conductor positions such that $z_{11}=z_{22}$ and $y_{11}=y_{22}$. In such a case, Equations (3) and (4) become

(7) $$\begin{array}{c} \frac{d^{2}}{dx^{2}}\left[\begin{array}{c} V_{1}\\ V_{2} \end{array}\right]=\left[\begin{array}{cc} P_{1} & P_{2}\\ P_{2} & P_{1} \end{array}\right]\left[\begin{array}{c} V_{1}\\ V_{2} \end{array}\right] \end{array}$$

and

(8) $$\begin{array}{c} \frac{d^{2}}{dx^{2}}\left[\begin{array}{c} I_{1}\\ I_{2} \end{array}\right]=\left[\begin{array}{cc} P_{1} & P_{2}\\ P_{2} & P_{1} \end{array}\right]\left[\begin{array}{c} I_{1}\\ I_{2} \end{array}\right], \end{array}$$

where

(9) $$\begin{array}{c} P_{1}=y_{11}z_{11}+y_{12}z_{12}\\ P_{2}=y_{11}z_{12}+y_{12}z_{11}. \end{array}$$

If a differential excitation is considered $I_{1}=-I_{2}$ and Equation (8) becomes

(10) $$\frac{d^{2}I_{1}}{dx^{2}}=\left(P_{1}-P_{2}\right)I_{1},$$

with a propagation constant

(11) $$\gamma_{d}=\sqrt{\left(z_{11}-z_{12}\right)\left(y_{11}-y_{12}\right)}$$

and characteristic impedance

(12) $$Z_{0}=\sqrt{\left(z_{11}-z_{12}\right)/\left(y_{11}-y_{12}\right)}.$$

## 4. Proposed Method for Identification and Location of Track Circuit Failures

In the TC, the rails can be seen as wires of a cable transmitting the information from the transmitter (battery) up to the receiver (relay). Therefore, the track can be modelled as a TL (see Section 3), and a discontinuity problem such as a broken rail or a short across the rails could be identified and located using the signal reflection properties [21].

The reflection coefficient of a TL can be written as

(13) $$\Gamma =\frac{Signal_{reflected}}{Signal_{incident}}=\frac{Z_{L}-Z_{0}}{Z_{0}+Z_{L}},$$

where $Z_{0}$ is the characteristic impedance of the TL and $Z_{L}$ is the impedance of the discontinuity. For instance, the reflection coefficient for an open circuit ($Z_{L}=\infty$) is 1 and the reflection coefficient for a short circuit ($Z_{L}=0$) is −1. The time or phase delay between the incident and reflected signals is related to the distance to the fault, and the observed magnitude of the reflection coefficient is related to the impedance of the discontinuity.

Due to the TC behavior as a TL, that was not designed for communication purposes imposing high losses for frequencies above the kHz range, techniques based on FDR are more suitable than the ones based on the time domain [25].

FDR sends a set of stepped-frequency sine waves down to the TL [26]. One of the simplest forms of FDR implementation is through Standing Waves Ratio (SWR). SWR systems measure the magnitude of the standing wave created by the superposition of the incident and reflected signals on the TL [27]. The sum of these two sine waves will have a series of peaks that are caused by their constructive interference and nulls caused by destructive interference [27]. As the frequency is swept, these nulls can be identified, or the behavior of the resulted curve (frequency × amplitude) can be used in order to identify and locate the discontinuity.

In SWR, the frequency of the sinusoidal signal is swept within a given bandwidth ($f_{1}$ through $f_{2}$) with step size Δf. It reflects back and is superimposed on the incident wave. The combination of the incident and reflected waves (standing wave) can be modeled as

(14) $$s\left(t\right)=A\left[sin\left(\omega t\right)+\Gamma e^{-(\alpha +j\beta )x}sin\left(\omega t\right)\right],$$

where s(t) is the observed signal in the injection side, *A* and ω are the amplitude and angular frequency (which is a scalar measure of rotation rate calculated by ω=2πf) of the injected signal, respectively, α is the attenuation constant per unit length of the TL, β is the phase constant per unit length of the TL, Γ is the reflection coefficient (see (13)) and *x* is two times the location of the impedance discontinuity.

The phase and attenuation constants of a MTL, considering the model in Figure 3, are defined by the propagation constant $\gamma_{d}$ in Equation (11), where

(15) $$\gamma_{d}=\alpha +j\beta =\sqrt{\left(z_{11}-z_{12}\right)\left(y_{11}-y_{12}\right)}.$$

**Figure 4:**
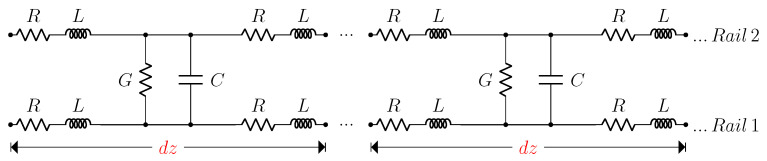
Lossy transmission line model with distributed parameters.

For the specific case of a two-wire lossy transmission line with differential injection, the model in Figure 4 can be used, where *R* is the line resistance per unit length, *L* is the line inductance per unit length, *C* is the capacitance per unit length between the two conductors and *G* is the line conductance per unit length between the two conductors. In this case, the propagation constant results in

(16) $$\gamma_{d}=\alpha +j\beta =\sqrt{\left(R+j\omega L)(G+j\omega C\right)}.$$

Equation (14) can be rewritten as

(17) $$Bsin\left(\omega t+\phi \right)=A\left[sin\left(\omega t\right)+\Gamma e^{-\alpha x}sin\left(\omega t-\beta x\right)\right],$$

where *B* is the amplitude of the observed signal s(t). Rewriting (17) as a sum of complex exponentials

(18) $$\begin{array}{c} \;\;\;\;\;\;\;\;\;\;\;\;\;\;\;\;\;\;\;\;\;\;\;\;\;\;\;\;\;\;\;\;\;\;\;\;\;\;\;\;\;\;\;\;B\left[\frac{e^{j(\omega t+\phi )}-e^{-j(\omega t+\phi )}}{2j}\right]=\\ A\left[\frac{e^{j\omega t}-e^{-j\omega t}}{2j}+\Gamma e^{-\alpha x}\frac{e^{j(\omega t-\beta x)}-e^{-j(\omega t-\beta x)}}{2j}\right] \end{array}$$

and rearranging the terms, follows

(19) $$\begin{array}{c} \;\;\;\;\;\;\;\;\;\;\;\;\;\;\;\;\;\;\;\;\;\;\;\;\;\;\;\;\;\;\;\;\;\;\;\;\;\;\;\;\;\;\;\;\;\;\;\;\;\;B\left[\frac{e^{j(\omega t+\phi )}-e^{-j(\omega t+\phi )}}{2j}\right]=\\ A\left[\frac{\left(1+\Gamma e^{-(\alpha +j\beta )x}\right)e^{j\omega t}-\left(1+\Gamma e^{-(\alpha -j\beta )x}\right)e^{-j\omega t}}{2j}\right]. \end{array}$$

Therefore, the amplitude *B* of the observed standing wave is given by

(20) $$B=A\left|1+\Gamma e^{-(\alpha +j\beta )x}\right|.$$

For lossless transmission lines (R=G=0), the attenuation α of the reflected signal can be neglected and the phase constant is $\beta =\omega \sqrt{LC}$, where *L* and *C* are the inductance and capacitance per unit length of the TL, respectively. Therefore, for hard faults in lossless transmission lines (normally in the kHz range), the accuracy of SWR is quite good and the null detection can be used to locate the discontinuity problem [25]. In this case, (20) can be rewritten as

(21) $$B=A\left|1+\Gamma e^{-j\omega \sqrt{LC}2D}\right|,$$

where *D* is the distance of the fault. In lossless transmission lines, the propagation velocity is $v=1/\sqrt{LC}$ and (21) can rewritten in terms of *v*

(22) $$B=A\left|1+\Gamma e^{-j\omega 2D/v}\right|.$$

It can be seen that the amplitude *B* is a function of the angular frequency ω, when it is stepped over a given range ($\omega_{1}$ through $\omega_{2}$), which is related to the frequency (ω=2πf).

**Figure 5:**
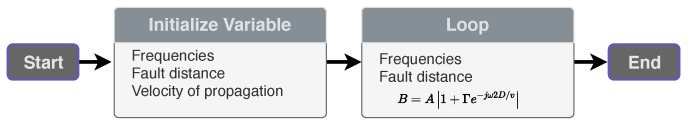
Diagram illustrating simulation for Equation (22).

**Figure 6:**
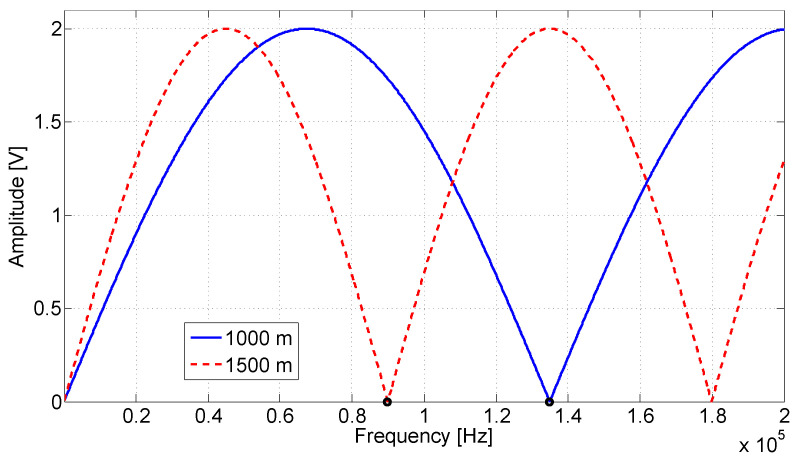
Behavior of the observed amplitude *B* with respect to the frequency as in (22). Short at 1000 m and 1500 m. The velocity of propagation *v* is considered as 90% the velocity of light and the frequency bandwidth is 100 Hz up to 200 kHz. The frequency was varied in steps of 100 Hz. The dots marked with a circle correspond to the identification of the null.

**Figure 7:**
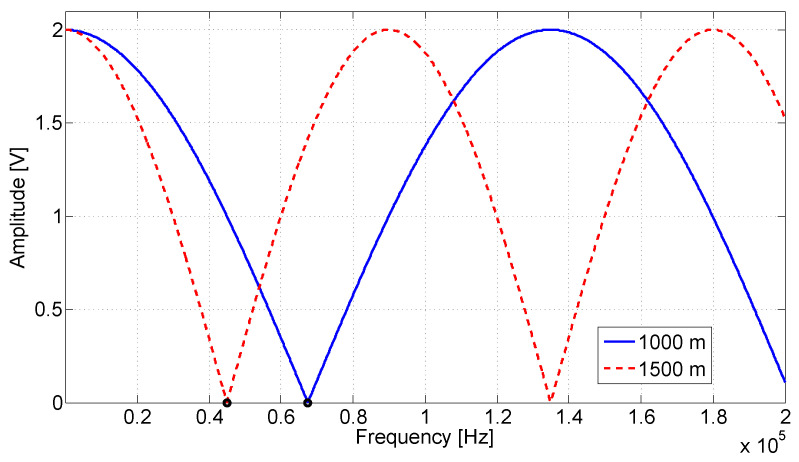
Behavior of the observed amplitude *B* with respect to the frequency as in (22). Open circuit at 1000 m and 1500 m. The velocity of propagation *v* is considered as 90% the velocity of light and the frequency bandwidth is 100 Hz up to 200 kHz. The frequency was varied in steps of 100 Hz. The dots marked with a circle correspond to the identification of the null.

Figure 5 presents an illustrating diagram simulation for Equation (22). The simulation was performed to illustrate the observed signal amplitude of *B* with respect to the frequency when short circuits are considered in two different distances, shorts at 1000 m and 1500 m. In the simulation, the velocity of propagation *v* was considered as 90% the velocity of light, the frequency bandwidth was 100 Hz up to 200 kHz and the frequency was varied in steps of 100 Hz. Figure 6 shows the simulation results, where is possible to verify that the frequency difference between the nulls is inversely proportional to the discontinuity distance and the convex behavior of the waveform indicates that it is related to a short circuit (Γ=−1). A similar simulation considering open circuits at 1000 m and 1500 m was performed and can be seen in Figure 7. It can be seen that for low impedance faults the curve grows in its initial part, while for high impedance faults the curve decreases. Therefore, it is possible to identify the fault, and its location can be estimated using the null detection technique.

**Figure 8:**
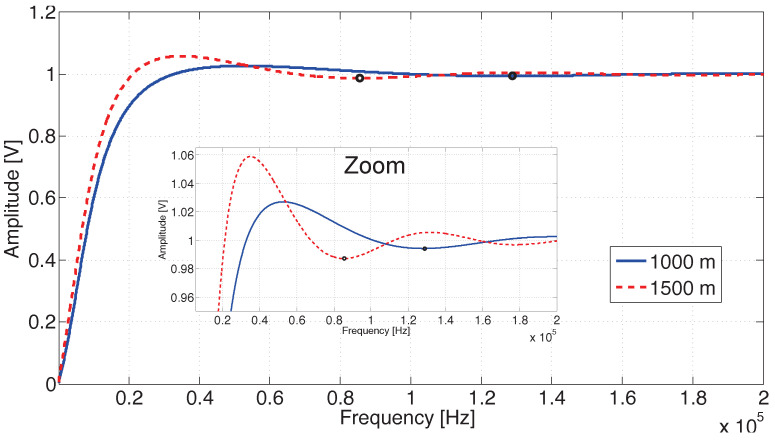
Behavior of the observed amplitude *B* with respect to the frequency for a lossy transmission line, as in (20). Short circuit at 1000 m and 1500 m. The velocity of propagation *v* is constant and considered as 90% the velocity of light and the frequency bandwidth is 100 Hz up to 200 kHz. The frequency was varied in steps of 100 Hz. The dots marked with a circle correspond to the identification of the null.

The use of a lossless TL for modeling the TC is not reasonable [28], therefore, the behavior of *B* with respect to the frequency is different from the one observed in Figure 6. A simplified simulation of Equation (20) for lossy transmission lines with low impedance faults at 1000 m and 1500 m was carried out, considering a first-order frequency-dependent attenuation for the reflected waveform and, for sake of simplicity, the propagation velocity *v* was considered constant as 90% the velocity of light. The result can be seen in Figure 8, where it is possible to verify the attenuation effect on the curve behavior in contrast to the behavior seen in Figure 6.

Figure 6 and Figure 8 were generated by simulation from Equation (22). They were considered a circuit containing a short circuit (Γ=−1), and the distances of 1000 m and 1500 m for fault. In Figure 6, the attenuation effects are ignored. The waveforms present successive peaks and nulls produced by the superposition of incident and reflected waves, which cause constructive and destructive interferences, respectively. Figure 8, the attenuation was considered, which reduced the amplitude of the peaks and valleys. In the zoom region shown by the figure, it is still possible to observe them. However, the contained attenuation quickly reduces the waveform oscillation and makes it difficult to apply null detection techniques.

**Figure 9:**
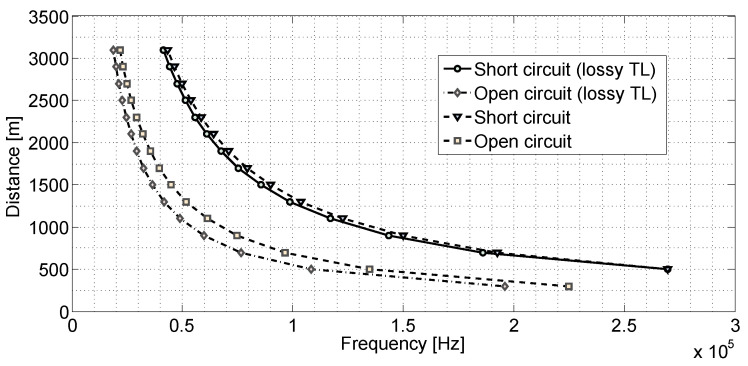
Distance from the failure obtained at the frequency of the first null of the amplitude waveform.

Figure 9 shows the relationship between the fault position and the frequency of the first null of the amplitude waveform obtained with Equation (22) simulation. In the figure, other fault positions were generated, and null detection was applied. It can be observed in Figure 6 the identification of the null point is marked with a circle. These marks are at 89,900 and 134,900 Hz frequencies, which correspond with null point detection in a short fault with a distance of 1500 m and 1000 m, respectively. The same analysis can be observed for Figure 7 and Figure 8. In them were also identified as the null points marked with a circle.

The demonstration presented in Figure 9, allows estimating the fault position by detecting null for the simulated equations. In this situation, with the obtained Fitting, it would be sufficient to correlate the frequency of the null point identified to estimate the failure’s position. In the simulation, the velocity of propagation *v* was considered as 90% the velocity of light, and the frequency bandwidth was 100 Hz up to 300 kHz.

Accurate measurements of lossy TL parameters of a TC is not a simple task [23,24,28] and could introduce large errors in the fault location estimation. Due to this reason, it is proposed for TC fault identification and location the application of the SWR technique based on the received signal amplitude × frequency curve, using the following procedure:

1. disconnect the track circuit power supply;
2. inject in one edge of the track circuit a sinusoidal signal with constant amplitude stepped over a certain frequency bandwidth;
3. for each injected sinusoidal, measure the amplitude of the waveform at the same point that the signal was injected;
4. build the signal amplitude × frequency curve;
5. the fault type can be identified through the curve behavior. If the amplitude starts growing, the fault is related to a low impedance between rails, otherwise, the fault is caused by a discontinuity problem in a rail joint or due to a broken rail;
6. the location can be estimated through a non-linear regression technique using as inputs the acquired amplitudes for a given frequency scan, avoiding the direct measurement of the track circuit parameters.

**Figure 10:**
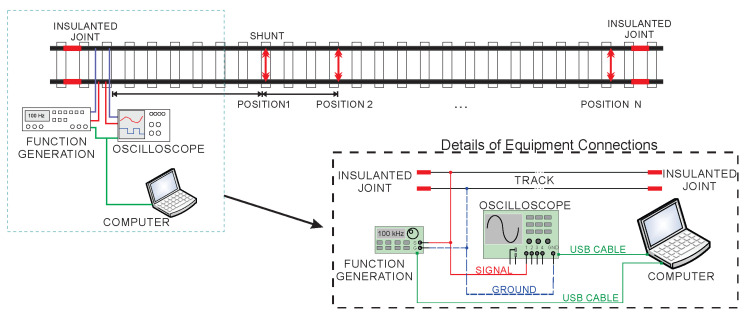
Connection of the proposed system with the track circuit.

The connection scheme of the proposed system on the TC can be seen in Figure 10. The system is basically composed of a sinusoidal source generation, a digital signal acquisition system and a processing and control device. The figure also shows the details of the connections between the equipment and the rails. The signal generator and oscilloscope are connected to the computer via a USB cable. The signal tip of the oscilloscope and the signal generator are connected on one side of the rails and the other the ground cables of both types of equipment. The software developed allows the adjustment of some parameters in the signal generator such as frequency and amplitude and the data storage process in the oscilloscope. It automates the actuation of the equipment and performs the frequency variation of the injected signal during the analysis.

## 5. Method Validation

In this paper, in order to validate the proposed method, a system was implemented using low cost laboratory equipments and a portable computer, as below:

- Signal Generator (*Agilent 33210A*): sinusoidal signal generation;
- Digital Scope (*Tektronix TDS 2024B*): signal reception and acquisition;
- Rugged Laptop (*DELL Latitude 14 Rugged Laptop*): control and processing.

**Figure 11:**
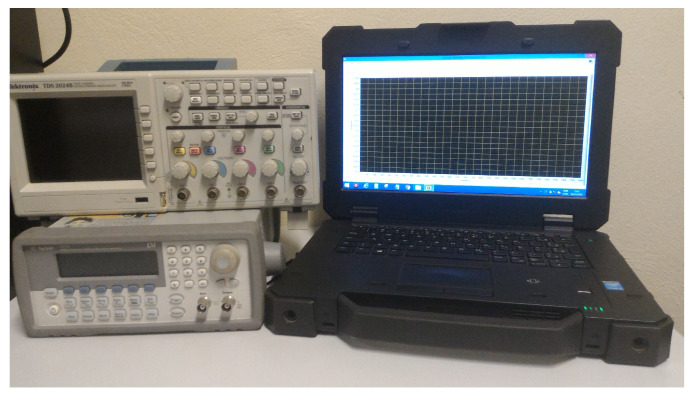
Implemented system for identification and location of track circuit faults.

The implemented system can be seen in Figure 11. A dedicated software was developed in order to control the signal injection, acquisition and to perform the required calculations.

An efficient implementation of the system can be done using:

- A voltage-controlled oscillator (VCO) for a signal generation;
- An amplifier, a receiver and a precision rectifier for signal conditioning;
- An analog to digital converter (ADC);
- A processing unit composed of a microcontroller or Field Programmable Gate Array (FPGA).

**Figure 12:**
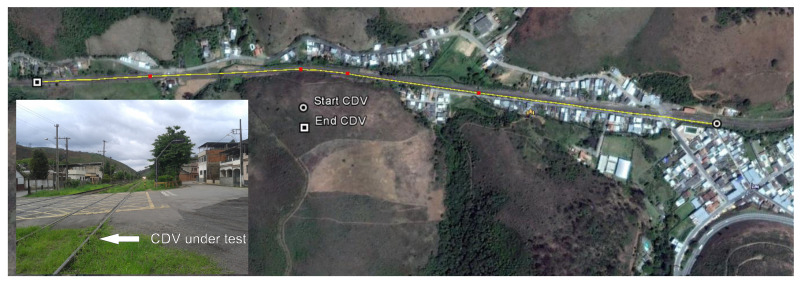
Satellite image of the track circuit under test from the MRS railway network, located at Ewbank da Câmara, MG, Brazil.

Envisaging the validation of the proposed method for identification and localization of TC faults, a field test was carried out at the MRS rail network in Brazil. A TC with approximately 1500 m long was used, located at Ewbank da Câmara, Minas Gerais. Figure 12 is a satellite image showing the TC under test. The proposed system was connected in the opposite end of the TC with respect to the relay and the TC voltage source was disconnected from the rails to avoid interference.

Low impedance faults were applied in different locations (150, 200, 300, 400, 500, 600, 700, 800, 1000, 1100, 1200, 1300, 1400 and 1500 m) of the TC using a shunt device (a low resistance cable with two connectors at the ends that can be fixed on both rails of the TC, acting as a low impedance path between the rails) and for each location, the frequency scan was performed and the peak to peak (PTP) value of the waveform was acquired and stored. Due to practical limitations, it was not possible to brake a rail or to simulated a discontinuity problem in order to test the system for high impedance faults. The injected sinusoidal PTP voltage (the *Agilent 33210A* output resistance is 50 Ω) was 20 V, the frequency scan was performed from 100 Hz up to 200 kHz and for each frequency three acquisitions of the PTP value of the waveform were stored. The frequency was stepped according to:

- 100 to 1000 Hz in steps of 100 Hz;
- 1000 to 10,000 Hz in steps of 500 Hz;
- 10,000 to 100,000 Hz in steps of 5000 Hz;
- 100,000 to 200,000 Hz in steps of 10,000 Hz.

**Figure 13:**
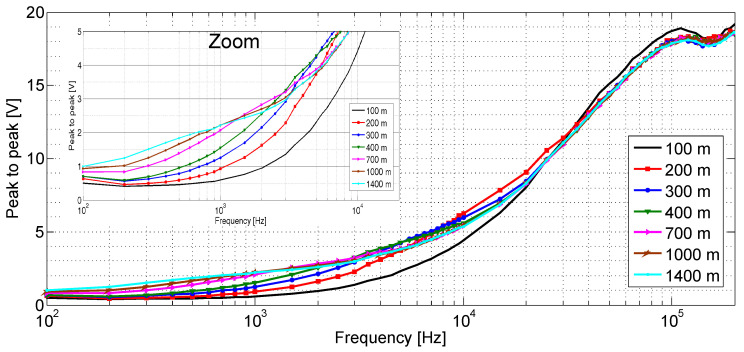
Observed sinusoidal peak to peak (PTP) value (Volts) with respect to the frequency (Hertz) for a track circuit in the MRS railway network for some short circuit positions. The frequency scan was from 100 Hz up to 200 kHz. The zoom was performed between frequencies from 0 to 6 kHz and amplitude from 0 to 5 PTP [V].

Figure 13 shows the observed PTP value of the acquired waveform with respect to the frequency for some faults. As expected, both curves are initially growing indicating that the problem is caused by a low impedance fault. Additionally, the figure shows a zoom region between 0 and 20 kHz on the abscissa axis and from 0 to 5 volts PTP for the ordinate axis. This region allows observing more easily the separation between the waveforms relative to the different distances of the faults, which can be used for fault location. Now in the region between the frequencies of 25 kHz up to 60 kHz and amplitude of 10 to 15 volts PTP the waveforms are very close to each other. This region containing the waveforms for different overlapping faults, but does not hinder the proposed method of nonlinear regression of performing the estimation of the position of the faults when analyzing a waveform.

**Figure 14:**
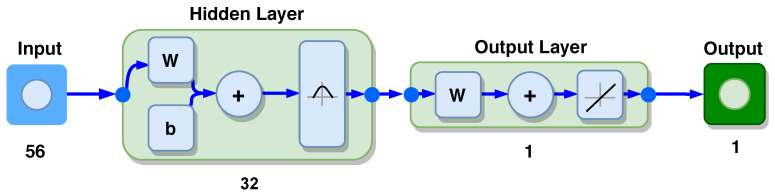
Structural diagram of the Generalised Regression Neural Network (GRNN). The network had 56 input and 32 hidden layers, where the normal distribution function was used as a probability density function. It has one output layer with a linear function.

It is worth to mention that the models and simulations presented in Section 4 did not take into account nonlinearities introduced by the system connection with TC such as power limitation of the sinusoidal signal generator, which is one of the main difficulties related to the accurate measurement of the TC parameters [28]. Therefore, as already mentioned in Section 4, in order to provide the fault location a non-linear regression technique can be applied using the amplitude values of the frequency scan as inputs. In this paper, a Generalised Regression Neural Network (GRNN) [29,30] is used to provide the fault location estimation. GRNN belongs to the category of probabilistic neural network that needs only a fraction of the training samples compared to the algorithms of a Backpropagation-based neural network [29,31]. It is widely applied in the parameter estimation process as can be seen in [32,33] works The network was trained using two acquisitions of the PTP value for each scan, leaving the third acquisition for validation. The GRNN input layer is composed by the PTP values of the frequency scan. Figure 14 represents the structure of GRNN applied in the process of estimating fault positions. The network had 56 input and 32 hidden layers, where the normal distribution function was used as a probability density function. It has one output layer with a linear function.

GRNN uses the normal distribution [34] as a probability density function, where each *X* training sample is used as the average of a normal distribution. The estimated output value $\hat{Y}$ can be viewed as a weighted average of all observed values [29]. Each output value of the $\left(Y_{i}\right)$ training samples is weighted exponentially according to the Euclidean distance from the *X* input sample to the $X_{i}$ training sample in order to get the $\hat{Y}$ estimate [32]. Therefore,

(23) $$\hat{Y}\left(X\right)=\frac{\sum_{i=1}^{n}Y_{i}\cdot e^{\left(-\frac{\Delta D_{i}^{2}}{2\sigma }\right)}}{\sum_{i=1}^{n}e^{\left(-\frac{▵D_{i}^{2}}{2\sigma }\right)}},$$

where σ is the spreading constant and ΔD is the distance between a training sample ($X_{i}$) for a prediction point (*X*).

The value of $\Delta D_{i}^{2}$ is calculated from the scalar function

(24) $$\Delta D_{i}^{2}={\left(X-X_{i}\right)}^{T}\cdot \left(X-X_{i}\right).$$

For small values of the distance ΔD, the term $e^{\left(-\Delta D_{i}^{2}/2\sigma \right)}$ becomes larger (tends to 1) and the contribution of the sample in the prediction is higher. For high distances, the term $e^{\left(-\Delta D_{i}^{2}/2\sigma \right)}$ becomes small and the training sample’s contribution to prediction is relatively low. For ΔD=0 the term $e^{\left(-\Delta D_{i}^{2}/2\sigma \right)}$ becomes 1 and the estimate perfectly represents the training sample [31].

The term $e^{\left(-\Delta D_{i}^{2}/2\sigma \right)}$ functions as the network activation function and acts as the weights for a given input. The spreading works as a smoothing parameter and can be adjusted during training [30]. The training process searches an optimal value of σ where the error is minimized. During the training stage, root-mean-square error (RMSE) was used, and 0.031 was determined how optimal.

(25) $$RMSE=\sqrt{\sum_{i=1}^{n}\frac{{\left(\hat{y_{i}}-y_{i}\right)}^{2}}{n}},$$

where $\hat{y_{i}}$ is predicted value, $y_{i}$ is observed value, and *n* is the number of observations.

**Figure 15:**
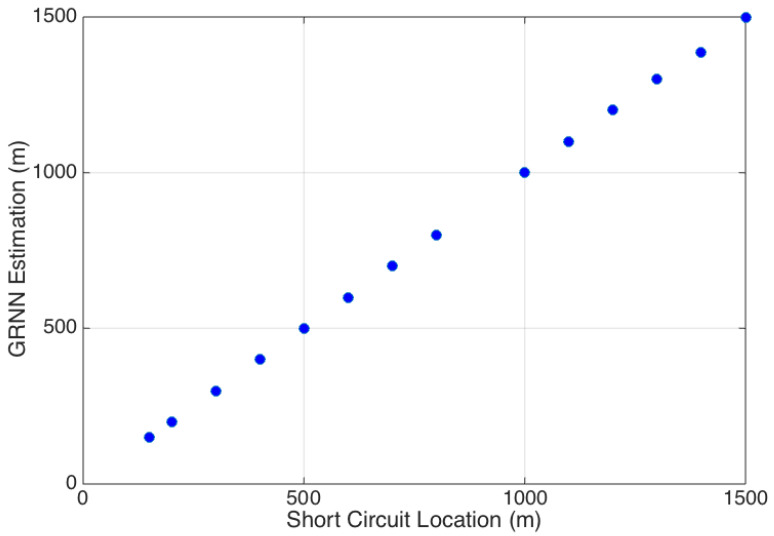
GRNN estimation results.

The results using the validation data (the third acquisition that was not used during the training phase) can be seen in Figure 15, where is possible to verify that the estimation values are very close to the actual fault location. The estimation error was negligible for all faults except for the one located at 1400 m, where the estimation was 1388 m (error of 12 m).

## 6. Discussion

The test performed at a TC of 1500 m long, from the MRS rail network, validated the proposed method for identification and localization of faults in track circuits. The method was able to identify and to provide estimations of the fault location with high accuracy. The fault location estimation was performed by a GRNN using only the amplitude values of the standing wave for the frequencies used in the scan (56 input nodes).

It is worth to mention that the null technique, commonly used in SWR, cannot be used in track circuits due to the lossy TL behavior. This fact introduces additional complexity for the system implementation in comparison to the null technique, but it is still possible to implement the proposed system in a similar way and with low cost.

The proposed method can be used for corrective maintenance, e.g., after the fault occurrence the maintenance team can use the system to identify and localize the fault, saving maintenance time, but it could be also embedded in the TC, modifying the TC components and providing new functionalities to it. The TC electronics could be modified, e.g., the voltage source and the relay can be replaced by the efficient system proposed in Section 4, providing continuous monitoring of the TC behavior, identification and localization of low or high impedance faults and an indication of train presence within the TC.

A limitation of the method is the need for calibration, which can result in a decreased accuracy in the estimation of location as a function of different TC, or of climatic conditions that affect the electrical parameters of the TC. Therefore to calibrate the system, it is necessary to obtain the standing wave’s amplitude curves as a function of frequency. These curves are obtained by performing tests on the TC, where faults for both short and open circuits must be reproduced. Differences in TC or climatic conditions that may affect the electrical parameters should be worked through tests in these regions to include these possible characteristics to the training bench.

Field testing, reproducing a short-rail failure is relatively easy, allowing more detailed characterization of TC electrical behavior in this situation. However, in the case of open-circuit reproduction is complex and requires time, because the process of disconnecting the rail and reconnecting is time-consuming and limits the use of the track.

As proposals for continuity of work, we have:

- The performance of field tests, where it is possible to make the rupture of the rail in several points of the TC, to allow the characterization and calibration of the system for broken rail conditions;
- To study the behavior of the system in coded TC, where there is the presence of pulses. These can generate a certain reduction in the precision of the method;
- Study the implementation of the method in an integrated way to the track circuit;
- For the case of the track circuit integrated system, evaluate the possibility of real-time monitoring of TC conditions and verify the feasibility of predictive methods of identifying failures;
- Study the possibility of the method resulting in a new type of TC.

## 7. Conclusions

In this paper, a new method for identification and localization of TC false occupancy faults based on reflectometry is proposed. Due to the TC behavior that imposes high losses for frequencies above the kHz range, FDR is used.

The proposed method relies on the behavior of the amplitude of the standing wave created by the superposition of the incident and reflected signals on the TC as the frequency is swept. The fault identification as a low impedance between rails or due to a discontinuity problem is straight forward, while the localization of the fault is performed by a generalized regression neural network using the amplitudes of the standing waves for a given scan. The estimation of the location of the failure point in the TC through non-linear regression of the standing wave amplitude curve as a function of frequency also constitutes an innovation about the reflectometry technique in the frequency domain.

The use of a GRNN to estimate the location of failures is especially advantageous due to its ability to converge with only a few available training samples, and also by presenting a rapid training process that converges to a global solution, avoiding local minimum points. The GRNN show good accuracy both for scenarios where simulations generated the data and for the data from field tests. The presence of regions within the waveform, where the measurements have a certain overlap, did not generate a problem for GRNN, which has a good generalization capacity.

A field test is carried out at the MRS rail network in a TC with approximately 1500 m. The results for low impedance fault identification and localization show high accuracy.

Therefore, this work presents a significant innovation by developing a new method for identifying and localizing false occupations in track circuits based on reflectometry in the frequency domain. The estimation of the failure point at TC through non-linear regression of the steady wave amplitude curve as a function of frequency is also an innovation in the technique of reflectometry in the frequency domain. The failures of the track circuits can cause significant economic losses to the railway system. Therefore, the contributions present to the area of electronic instrumentation and rail transport are incredibly relevant. A measurement system has been implemented to validate the proposed method, and field tests have been carried out. The achieved results showed that the method works with good accuracy and can be used to speed up and reduce railway companies’ maintenance costs.

Finally, the proposed method can be used for corrective maintenance, or it can be embedded in the TC, to provide it new functionalities.
